# Aortic Arch Pseudoaneurysm with Porcelain Aortic Arch and Triple-Vessel Coronary Artery Disease: Single-Stage Total Arch Replacement with Coronary Artery Bypass Grafting

**DOI:** 10.1055/a-2915-2888

**Published:** 2026-08-04

**Authors:** Harikrishnan Radhakrishnan, Amitabh Satsangi, Kanmaniyan Bala, Shruti Jobalia, Arindam Choudhury, Harsh Anand Singh

**Affiliations:** 1 Department of Cardiothoracic and Vascular Surgery 28730 All India Institute of Medical SciencesNew DelhiIndia; 2 Department of Cardiac Anaesthesia and Critical Care 28730 All India Institute of Medical SciencesNew DelhiIndia; 3 Department of Cardiovascular Radiology and Endovascular Interventions 28730 All India Institute of Medical SciencesNew DelhiIndia

**Keywords:** aortic arch pseudoaneurysm, porcelain aorta, coronary artery bypass grafting, total arch replacement, cerebral perfusion

## Abstract

Porcelain aortic arch with coronary artery disease poses a major surgical challenge. We report a 66-year-old man with a large arch pseudoaneurysm, circumferential arch calcification, left common carotid artery thrombosis, and triple-vessel coronary disease who underwent single-stage total arch replacement with coronary artery bypass grafting. Surgery utilized axillary and femoral cannulation, hypothermic circulatory arrest, and selective antegrade cerebral perfusion. Complete recovery followed a transient postoperative neurological deficit, demonstrating feasibility of tailored open repair.

## Introduction

Porcelain aorta, characterized by extensive circumferential calcification, significantly increases the risk of embolic complications during cardiac surgery. Involvement of the aortic arch further complicates management due to the need for cerebral protection. When associated with multivessel coronary disease, conventional surgical strategies may not be feasible, necessitating complex approaches such as combined arch replacement with coronary artery bypass grafting (CABG).

## Case Presentation

### Patient Presentation

A 66-year-old retired man presented with complaints of exertional chest pain for 2 months, corresponding to New York Heart Association class II. There was no history of syncope. His past medical history was significant for percutaneous coronary intervention to the left circumflex artery (LCX) in 2015 (records unavailable), long-standing hypertension (20 years), and type 2 diabetes mellitus (15 years). There was no prior history of cerebrovascular accident.

On clinical examination, the patient was hemodynamically stable with a heart rate of 68 beats/min and blood pressure of 148/78 mm Hg. Oxygen saturation was 100% on room air. Notably, the left carotid pulse was absent, whereas the right carotid and all peripheral pulses were palpable. Cardiovascular examination revealed a normally placed apex beat in the left fifth intercostal space at the anterior axillary line, with normal first and second heart sounds and no audible murmurs. Respiratory, abdominal, and neurological examinations were unremarkable.

### Diagnostic Workup

**Fig. 1:**
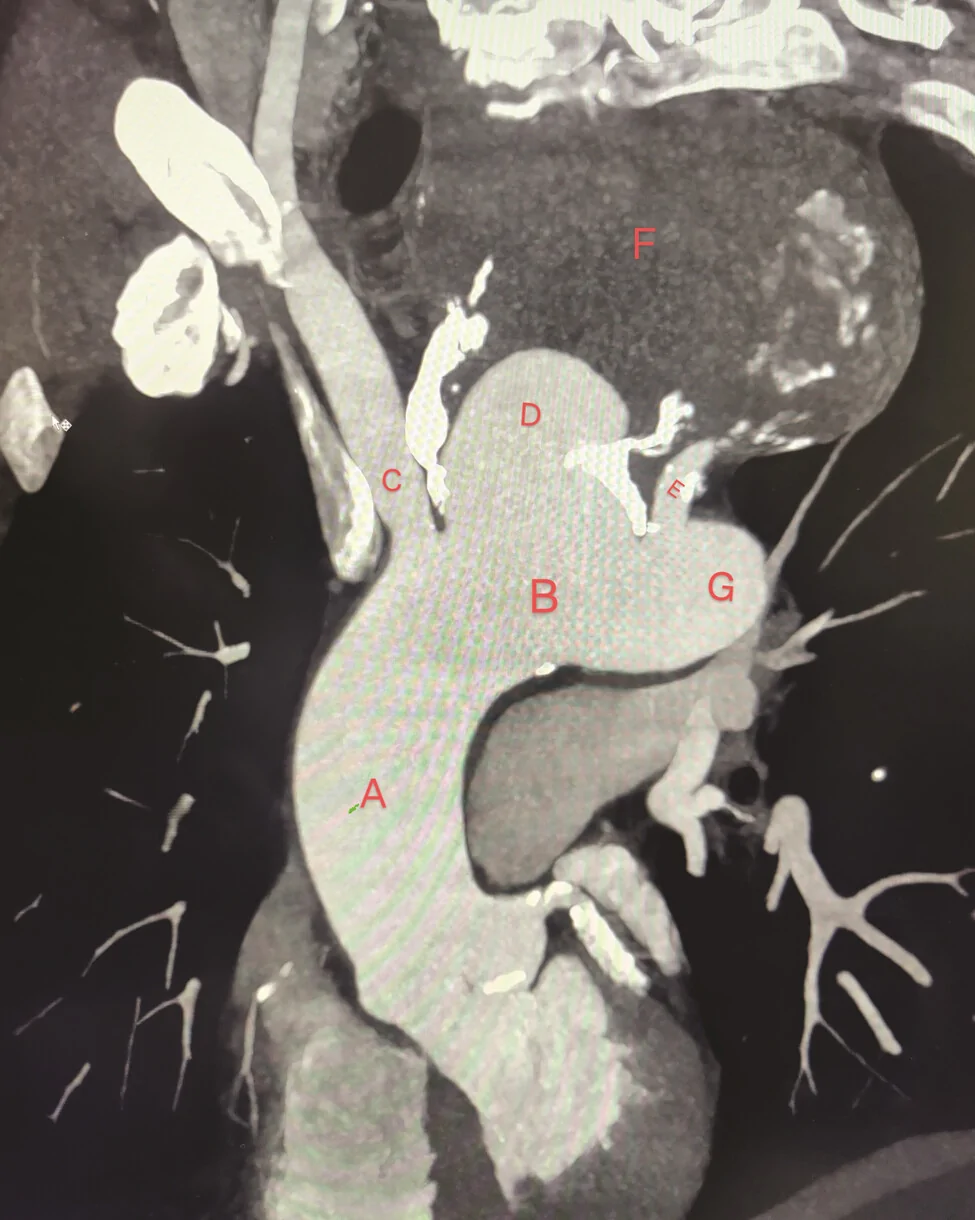
CT aortogram—aortic arch pseudoaneurysm with peripheral calcification (porcelain arch) and partial thrombosis. Labels: A, ascending aorta; B, aortic arch; C, innominate artery; D, pseudoaneurysm; E, left subclavian artery; F, thrombosed lumen; G, distal arch. CT, computed tomography.

**Fig. 2:**
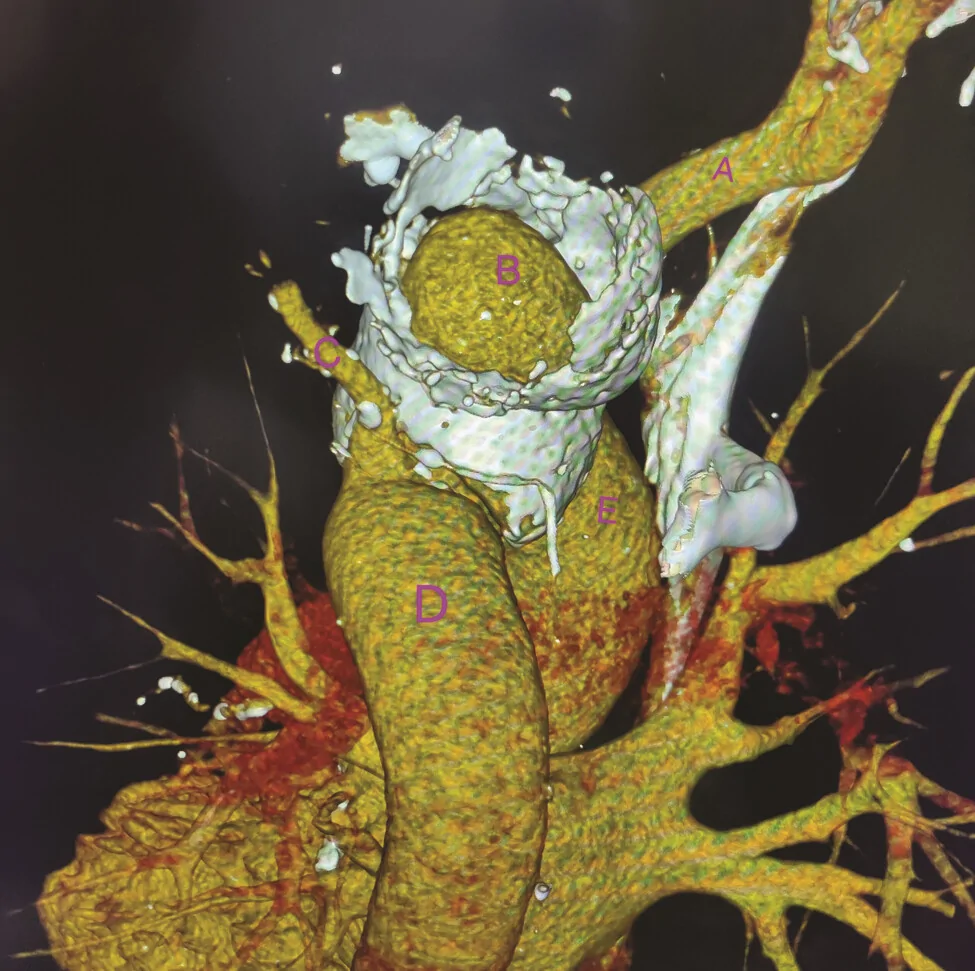
Volume-rendered three-dimensional CT reconstruction—the calcified aortic arch pseudoaneurysm and its relation to the arch vessels. Labels: A, innominate artery; B, pseudoaneurysm; C, left subclavian artery; D, distal arch; E, ascending aorta. CT, computed tomography.

**Fig. 3:**
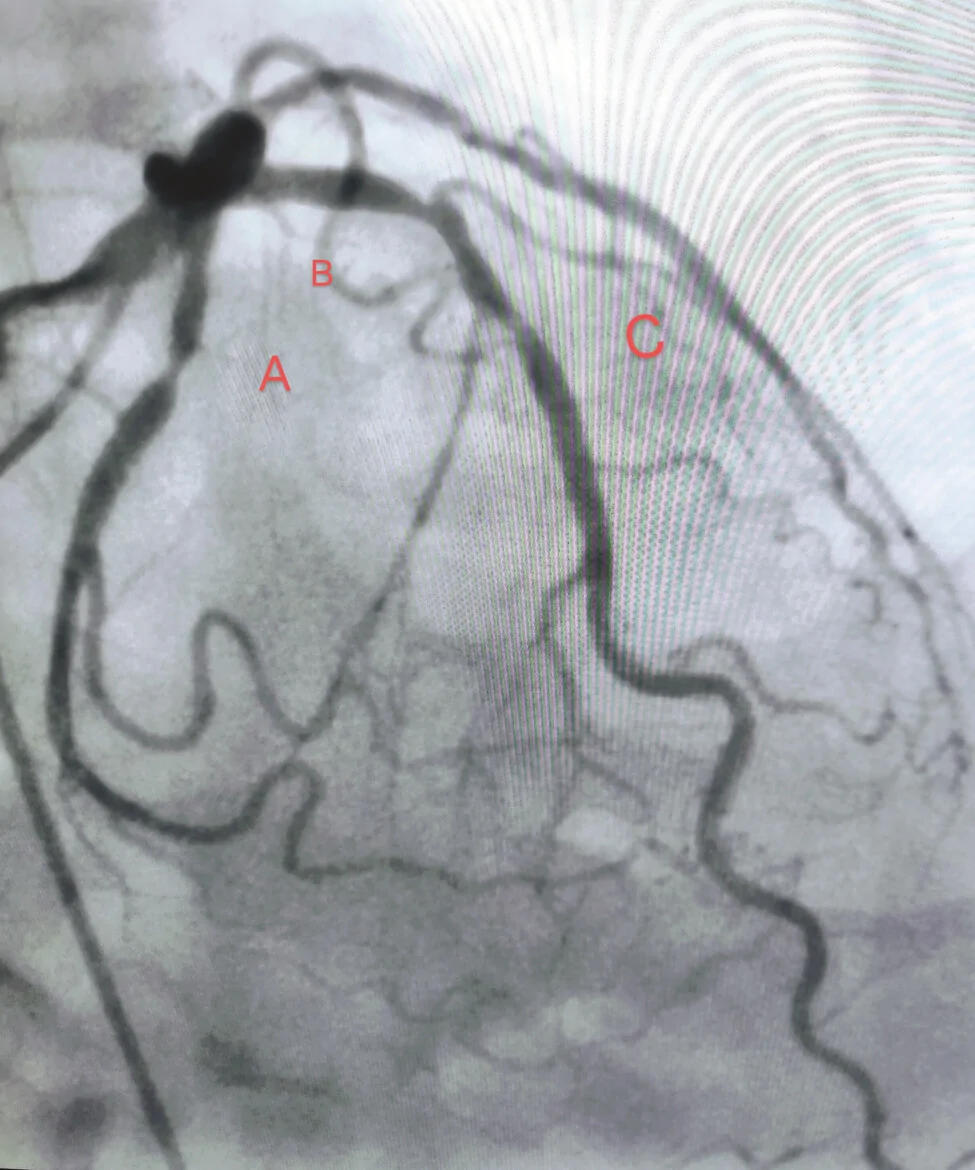
Coronary angiography—stenoses in the left anterior descending and left circumflex coronary arteries. Labels: A, LCX stenosis; B and C, LAD stenoses. LAD, left anterior descending artery; LCX, left circumflex artery.

Chest radiography demonstrated a large irregular mediastinal opacity involving the left upper mediastinum. Electrocardiogram-gated computed tomography aortography demonstrated a large saccular pseudoaneurysm arising from the superior aspect of the aortic arch between the innominate artery and left subclavian artery origins ( Figs. 1 and 2 ). The proximal and mid portions of the left common carotid artery were stenosed. Coronary angiography revealed triple-vessel coronary artery disease (CAD) with significant stenoses involving the left anterior descending (LAD), LCX, and right coronary arteries ( Fig. 3 ). Transesophageal echocardiography demonstrated preserved biventricular function without valvular abnormality or aortic root pathology.

### Operative Technique

**Fig. 4:**
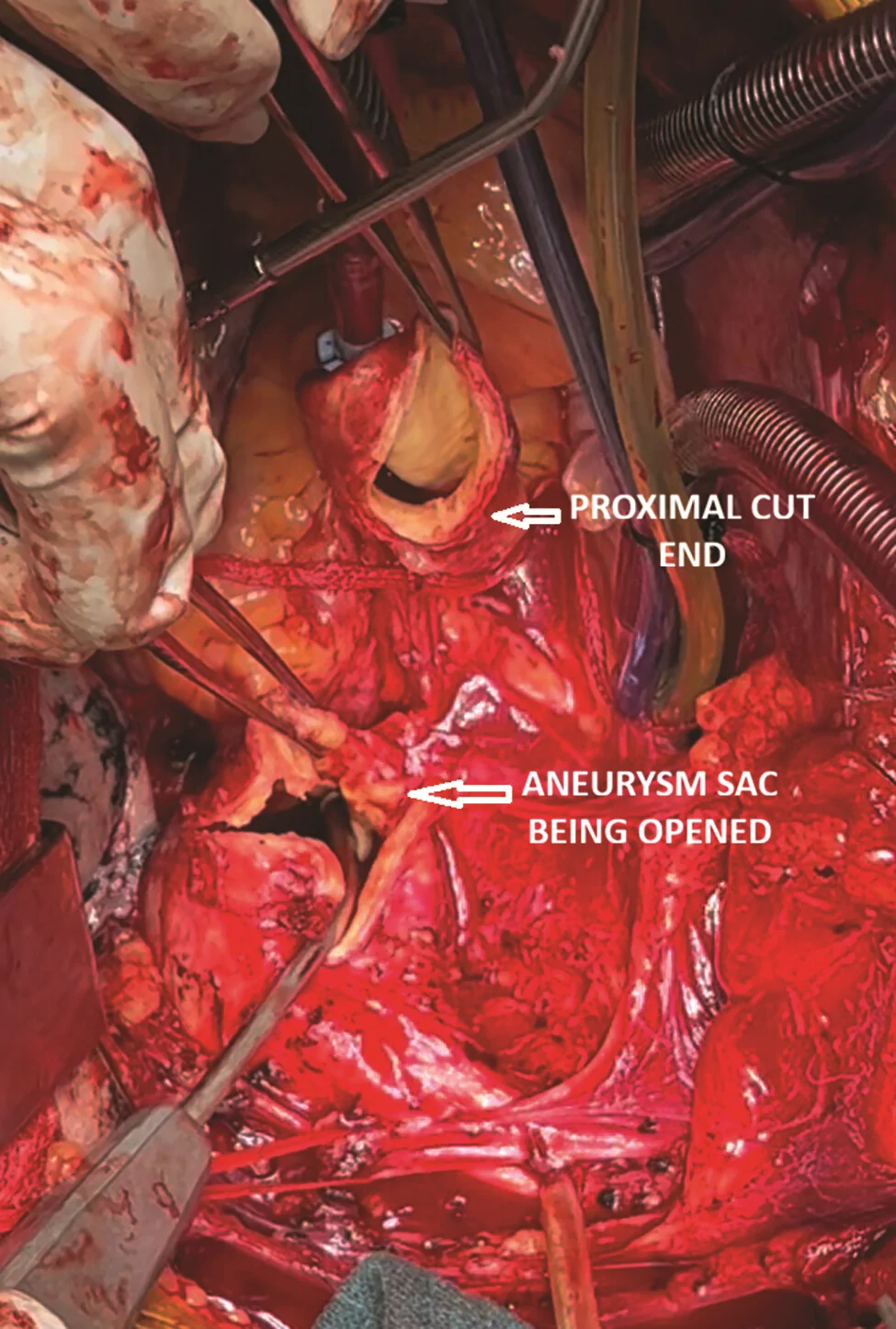
Intraoperative image—the opened aortic arch and pseudoaneurysm during total arch replacement under circulatory arrest with selective antegrade cerebral perfusion.

**Fig. 5:**
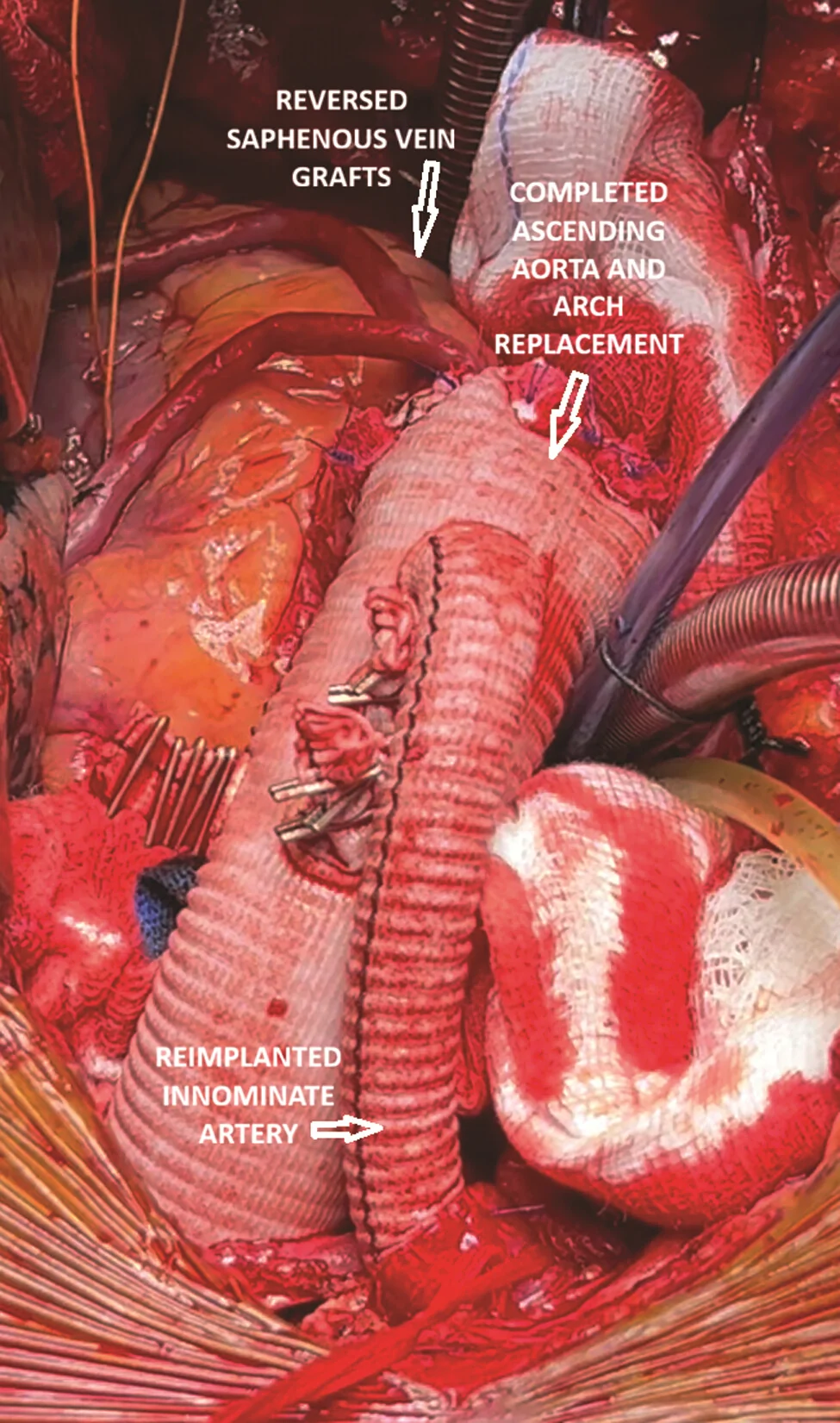
Completed total arch reconstruction and coronary artery bypass grafting.

The patient underwent single-stage total aortic arch replacement with concomitant CABG under moderate hypothermic circulatory arrest. Del Nido cardioplegia were used. Cardiopulmonary bypass was established using right axillary and femoral arterial cannulation with bicaval venous drainage. Selective antegrade cerebral perfusion (SACP) was provided via the axillary artery. Coronary revascularization was performed using saphenous vein grafts to LAD and obtuse marginal artery. The diseased aortic arch was excised and replaced with a branched Gelweave graft (26 × 8 × 8 × 8 mm). The innominate artery was reimplanted, and the left subclavian artery was ligated due to heavy calcification ( Fig. 4 and 5 ). The patient was extubated on postoperative day 3. He developed left upper limb monoparesis, with magnetic resonance imaging showing multiple cerebral infarcts. Conservative management led to gradual neurological recovery. He was discharged on postoperative day 29 in stable condition. At 3-month follow-up, he was asymptomatic with complete neurological recovery.

## Discussion

Aortic arch pseudoaneurysm is a rare but life-threatening condition resulting from a contained rupture of the aortic wall. Unlike true aneurysms, pseudoaneurysms are prone to rapid expansion and rupture. They most commonly arise secondary to atherosclerosis, prior surgical or catheter-based interventions, infection, or trauma. In the present case, the coexistence of a large saccular pseudoaneurysm, a heavily calcified (“porcelain”) aortic arch, and multivessel CAD represented a particularly complex surgical challenge. Porcelain aorta, characterized by extensive circumferential calcification of the aortic wall, significantly increases the risk of embolic stroke during cardiac surgery due to manipulation of friable atheromatous plaques. It limits conventional strategies such as aortic cross-clamping, cannulation, and proximal graft anastomosis, necessitating alternative approaches including peripheral cannulation, no-touch techniques, or hybrid/endovascular procedures.

In the expert consensus document by Czerny et al on thoracic aortic pathologies involving the aortic arch, various arch pathologies and management strategies are described; however, porcelain aorta receives no specific mention, highlighting the need for individualized management in such uncommon scenarios. 1

The choice of arterial cannulation and cerebral protection was critical in this patient. Thrombosis of the left common carotid artery rendered cerebral perfusion largely dependent on the right carotid circulation. Right axillary artery cannulation was therefore selected to facilitate antegrade cerebral perfusion, a strategy associated with lower neurological complications than deep hypothermic circulatory arrest alone. Spielvogel and Tang described a protocol employing perfusion pressures of 40 to 60 mm Hg, flow rates of 6 to 10 mL/kg/min, perfusate temperatures of 20 to 28°C, α-stat pH management, hematocrit of 25 to 30%, near-infrared spectroscopy (NIRS) monitoring, and bilateral perfusion when prolonged SACP is anticipated. 2 In our case, perfusion pressures were maintained within this range, moderate hypothermia was used, and cerebral perfusion was monitored using preoperative assessment of the circle of Willis and intraoperative NIRS.

A single-stage total arch replacement (TAR) with CABG was performed. Although hybrid approaches such as supra-aortic debranching followed by thoracic endovascular aortic repair are increasingly utilized, especially in high-risk patients, open single-stage repair remains the gold standard in anatomically unsuitable cases, as discussed by Preventza et al. 3 This approach avoids interval rupture and allows definitive treatment of both pathologies during a single procedure.

Imasaka et al, in a comparative study of 157 patients undergoing TAR with and without CABG, reported that although prolonged cardiopulmonary bypass time was associated with increased mortality, concomitant CABG could be performed safely with favorable outcomes. 4 Similarly, Okada et al reported hospital mortality rates of 1.5% for isolated TAR and 7.2% for TAR with CABG, emphasizing the greater comorbidity burden in patients requiring concomitant coronary revascularization. 5

Neurological complications remain a major concern in arch surgery, with reported stroke rates of 5 to 15%. 6 This risk is further amplified in patients with porcelain aorta and cerebrovascular disease. Despite meticulous neuroprotective measures, our patient developed postoperative left upper limb monoparesis secondary to multiple cerebral infarcts. Gradual recovery with antiplatelet therapy and physiotherapy underscored the potential for meaningful neurological improvement.

Management of the left subclavian artery also required consideration. Although revascularization is generally recommended, ligation may be necessary when the vessel is heavily calcified or unsuitable for reconstruction. Adequate collateral circulation through the vertebral and internal thoracic arteries may provide satisfactory perfusion in selected patients, as observed in this case.

Single-stage TAR with concomitant CABG is a feasible option in selected patients with porcelain aortic arch and complex CAD when hybrid or endovascular approaches are unsuitable. Open surgical repair with tailored cerebral protection strategies remains the definitive treatment in anatomically unsuitable cases for endovascular therapy. Despite advances in surgical techniques, neurological complications remain a significant concern, underscoring the need for careful perioperative management and multidisciplinary care.
